# Evaluating Pedicled Pectoralis Major Myocutaneous Flap Reconstructions for Oral Malignancy and the Influence of Laterally Based Rotational Advancement Flap on Donor Site Integrity and Nipple Position in Male Patients

**DOI:** 10.7759/cureus.58022

**Published:** 2024-04-11

**Authors:** Gaurav Chaturvedi, Ajit K Kushwaha, Rohit Jha, Kanika Suhag

**Affiliations:** 1 Burns and Plastic Surgery, All India Institute of Medical Sciences, Bhopal, Bhopal, IND; 2 Surgical Oncology, Rajendra Institute of Medical Sciences, Ranchi, IND; 3 Anaesthesia, Bhopal Memorial Hospital and Research Centre, Bhopal, IND

**Keywords:** rotational advancement flap, nipple areolar complex, wide local excision, oral malignancy, pmmc flap

## Abstract

Oral cancer poses a significant health burden, particularly in the male population of India. This study focuses on evaluating the outcomes of 48 pedicled Pectoralis major myocutaneous (PMMC) flap reconstructions in male patients with oral malignancy. Given the challenges associated with microvascular flap reconstructions, especially in advanced cancer cases, older patients, and resource-constrained settings, the PMMC flap still serves as a valuable alternative.

The study introduces a novel approach by incorporating a laterally based rotational advancement flap (LBRA) to address donor site integrity and decrease the nipple-areolar complex (NAC) displacement. Traditionally, PMMC flap designs tend to cause inward shifting of the NAC during chest donor site closure, impacting the aesthetic outcome.

Surgical techniques involved wide local resection, neck dissection, and PMMC flap reconstruction. The Flap design included a horizontal orientation with adjustments based on defect location. Additionally, a laterally based rotational flap from the chest aided in donor site closure.

Results demonstrate the versatility and reliability of PMMC flap reconstructions, with no total flap necrosis or major complications observed in the 48 cases. The LBRA technique effectively mitigated NAC displacement. The study contributes to the existing literature by providing insights into the advantages of PMMC flap reconstructions and introducing a technique to optimize donor site closure and decrease the medial shifting of the nipple. The adaptability, reliable vascular supply, and simplified learning curve make the PMMC flap a preferred choice in resource-constrained settings with high patient demand.

In conclusion, this research underscores the continued relevance and effectiveness of the PMMC flap in head and neck reconstruction, offering satisfactory cosmetic and functional results. The introduction of the LBRA technique adds a nuanced dimension to improve outcomes, particularly in male patients with oral malignancy.

## Introduction

Oral cancer is a common cancer in the male population of India, accounting for 10.3% [1] of new cases every year. In our center, 16% of patients present with oral malignancy, of which the majority are in advanced stages of cancer and from a weaker socio-economic section with a history of chewing tobacco for years. In such patients, the pedicled pectoralis major myocutaneous (PMMC) flap plays a major role in reconstruction. The results of reconstruction by microvascular flaps are well proven to be superior, but in advanced cases of cancer, older patients, and patients with comorbidities, the failure rates of these flaps increase. Besides this, cost constraints, heavy patient load, and the availability of trained staff in microsurgery are also limiting factors [2-4].

Plastic surgeons commonly employ a vertical to-oblique orientation while designing the PMMC flap, but this approach can lead to inward shifting of the Nipple Areolar Complex (NAC) and tension during the closure of wider flaps on the chest donor site.

This research aimed to assess outcomes from 48 pedicled pectoralis major myocutaneous (PMMC) flap procedures in male patients while also exploring the use of a laterally based rotational advancement (LBRA) flap from the pectoral region to address donor site defects. The study investigated how incorporating supplementary skin from the chest's lateral aspect aided in closing larger donor site defects and mitigated NAC displacement.

## Materials and methods

This study was conducted at the Rajendra Institute of Medical Sciences (RIMS), Ranchi, one of the largest tertiary care centers in the eastern part of India, after getting approval from an ethical committee. This retrospective analysis involves 48 male patients who underwent surgical procedures for the removal of malignant lesions in the head and neck region or affecting the oral mucosa. The surgeries, which took place between June 2019 and February 2020, aimed to address skin or mucosal defects resulting from the excision. The operating surgeons were the same in all cases. All of these patients underwent wide local resection of the tumor and neck dissection with a PMMC flap cover. The data was collected from the IP and OP charts, patient photographs, and patient OPD follow-up records. Information retrieved includes demographic profile, clinical findings, site of the tumor, defect size, pedicle PMMC flap design and size, its reach, donor site closure by a laterally based skin flap, and displacement of the NAC (nipple-areolar complex). 

Surgical technique

**Figure 1:**
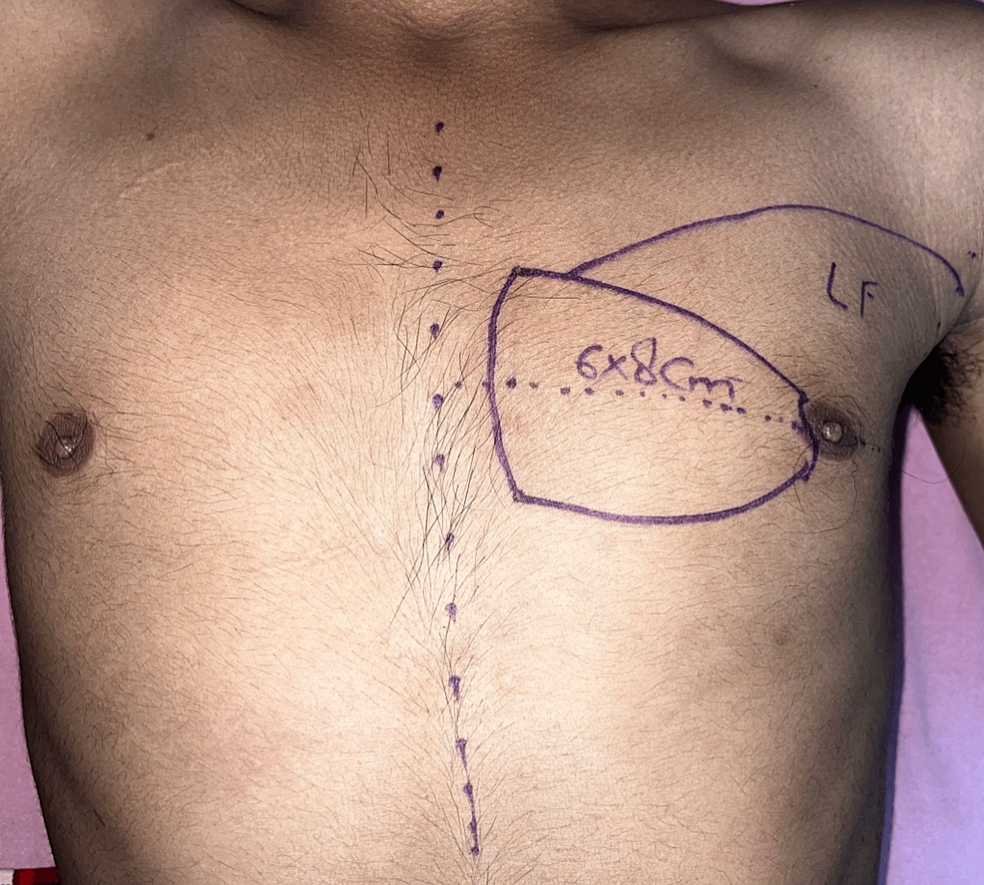
Preoperative marking of the PMMC flap along with the LBRA flap. Flap marking: The flap’s long axis is oriented horizontally, PMMC: Pectoralis major myocutaneous flap, LBRA/LF: Laterally based rotational advancement flap

Patients with histologically proven cases of malignancy were explained regarding the surgical procedure and the flap required to cover the defect. The cases were managed with resection involving wide local excision of the primary tumor +/- segmental mandibulectomy and neck dissection. Planning in reverse of the flap for assessing its reach to the defect site was done after removing the shoulder pillow used for neck extension. Before surgery, the distance from the midline of the chest (an imaginary line drawn from the suprasternal notch to the xiphisternum) to both sides of the nipples was measured horizontally as well as vertically from the mid-clavicular point to the superior end of the nipple. After surgery, the distance of the nipple on the operated side was measured again. The intraoperative flap was always designed horizontally over an imaginary line passing from the center of the nipple to include the skin perforator [5]. The superior and inferior markings of the flap can be adjusted downwards or upwards from this imaginary line, depending upon the location of the defect. Another curvilinear line marking extending up to the anterior axillary region was made from the superomedial end of the designed flap (Figure 1). The curvilinear marking delineates the flap originating from the lateral side of the chest, facilitating the rotational advancement of skin from the lateral aspect to the anterior aspect.

**Figure 2:**
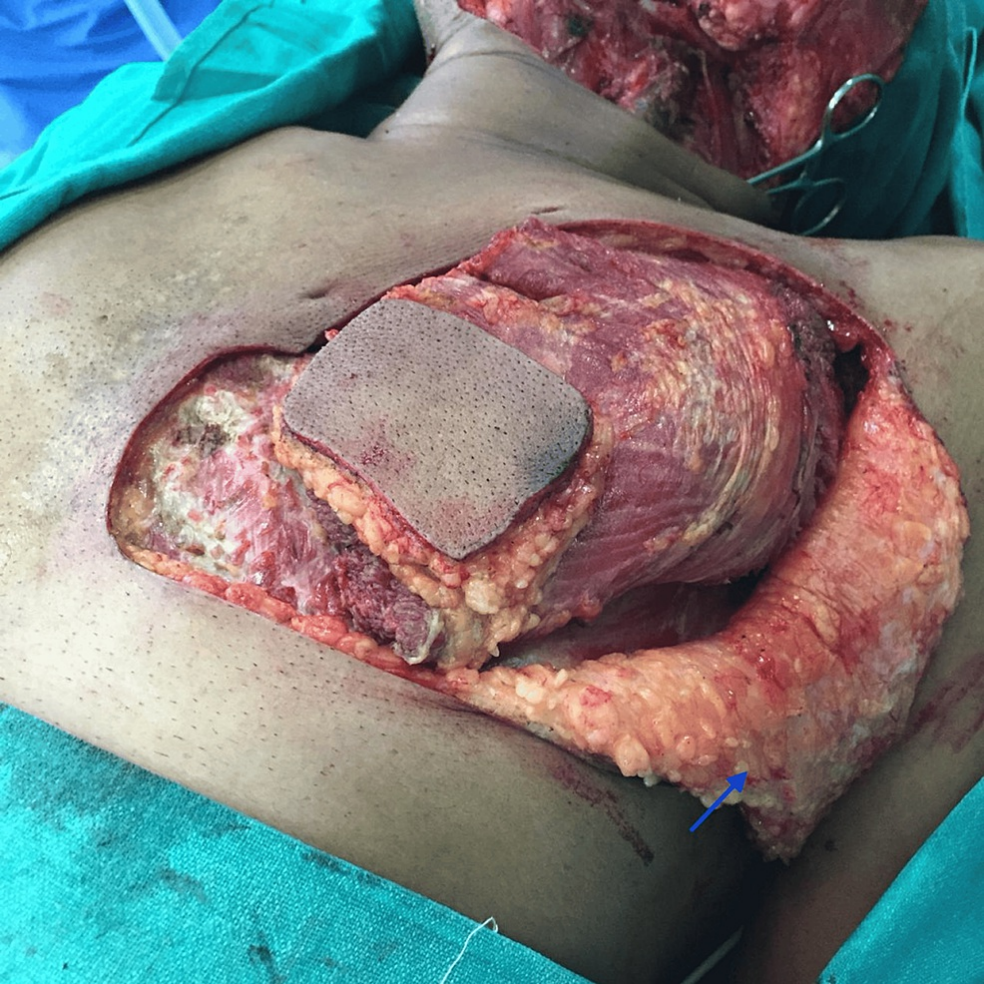
Case 1: PMMC flap and LBRA flap elevation. Flap elevation in a 53-year-old male. The skin island of the PMMC flap and the undersurface of the elevated laterally based rotational advancement flap are shown by the blue arrow.

The first incision was made over the curvilinear line, followed by an incision over the superior and lateral borders of the marked flap. The dissection was deepened until the pectoralis major (PM) muscle became visible and then extended laterally to elevate the laterally based skin flap (LBRA) and visualize the lateral border of the PM muscle. LBRA was then reflected laterally, and dissection was then continued between the pectoralis major and minor muscle planes. Exposure to the lower border of PM muscle can be enhanced by making a skin incision over the inferior border and further retracting the already elevated skin flap laterally. PM muscle can now be elevated from its lower border upwards after completing the incision on its medial side (Figure 2). The reason for making the medial incision at last was to prevent inadvertent shearing of the skin perforator. Except in the bi-paddle flap design, we have sacrificed the lateral pectoral artery supplying the muscle in each case to avoid tension and to increase the reach of the harvested flap. 

**Figure 3:**
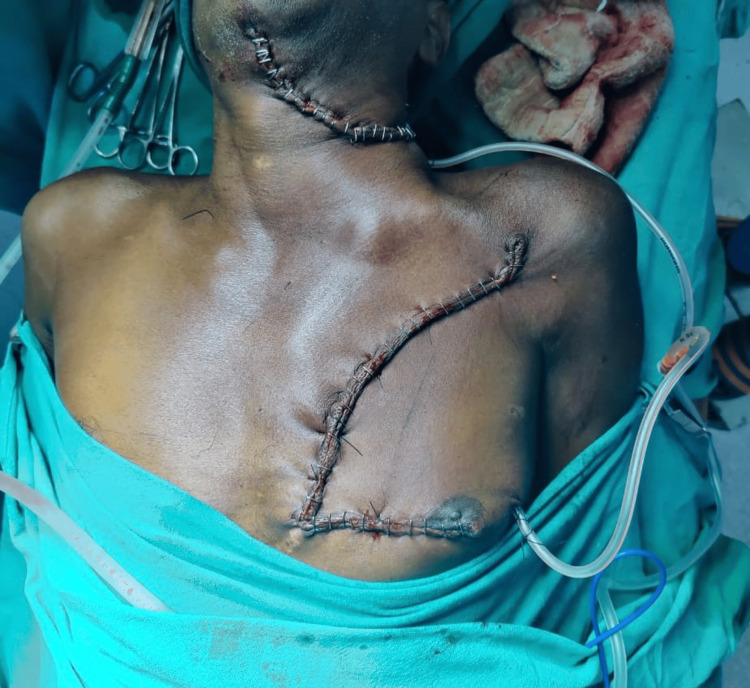
Closure of the donor site. Left side harvested PMMC flap donor side closure by laterally based rotational advancement flap.

**Figure 4:**
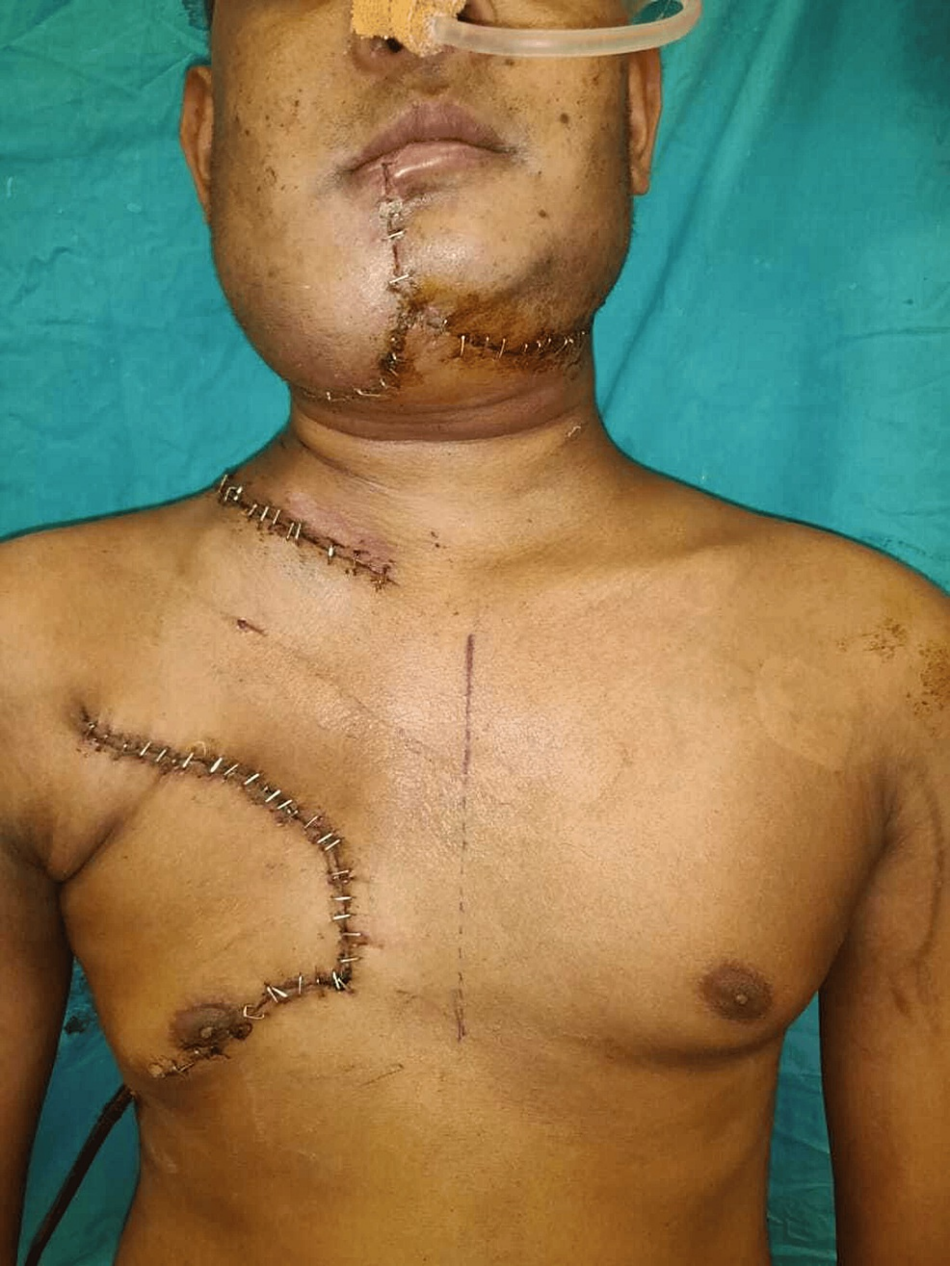
Case 2: Post-op image of the donor side PMMC was harvested, and closure of the donor side was done by using the LBRA flap. Minimal shifting of the nipple can be noted.

After PMMC flap harvesting and in-setting, the raised LBRA at the donor site was advanced and rotated to cover the donor site defect (Figure 3). Before skin closure at the donor area, the hemostasis sutures were placed at the cut end of the pectoralis muscle. After the surgery, the distance of the nipple on the operated side was measured again (Figure 4). 

**Figure 5:**
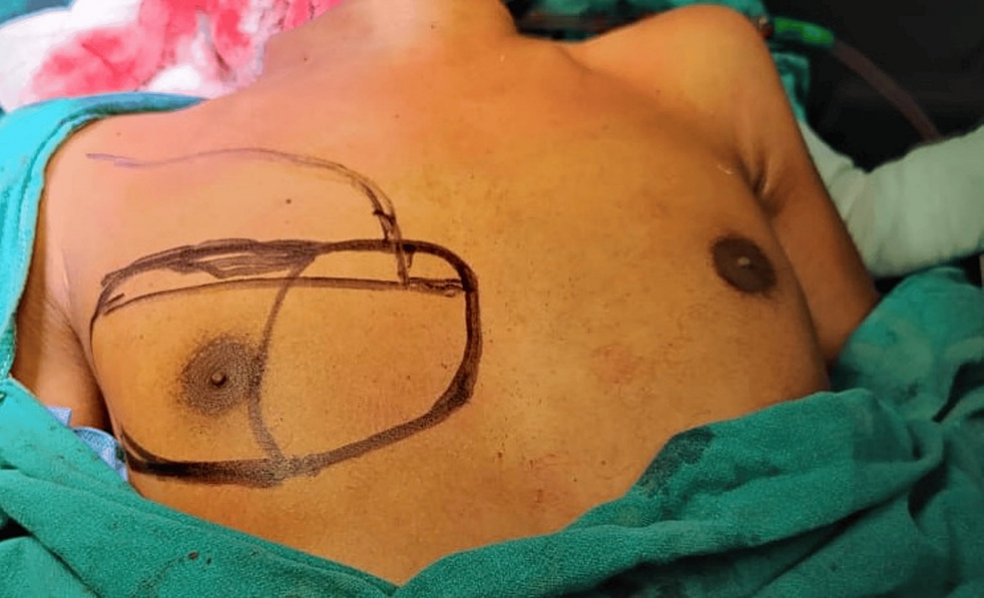
Marking of the Bipaddle PMMC flap The harvested flap's NAC was removed before its insetting. The inner half is designated for mucosal lining, while the outer half is utilized for outer lining purposes.

**Figure 6:**
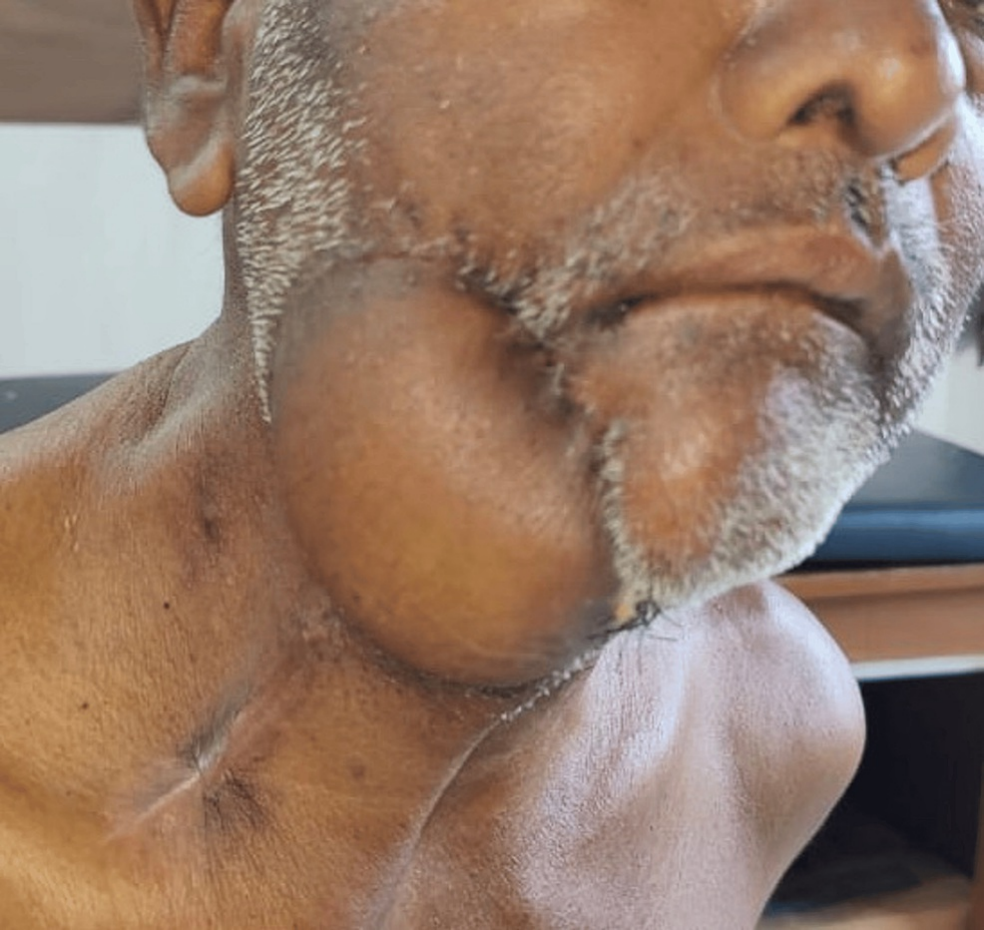
Follow-up image of the Bipaddle PMMC flap. A bipaddle PMMC flap is used in reconstructing the outer lining of the lower cheek and the right side lateral aspect of the lower lip.

**Figure 7:**
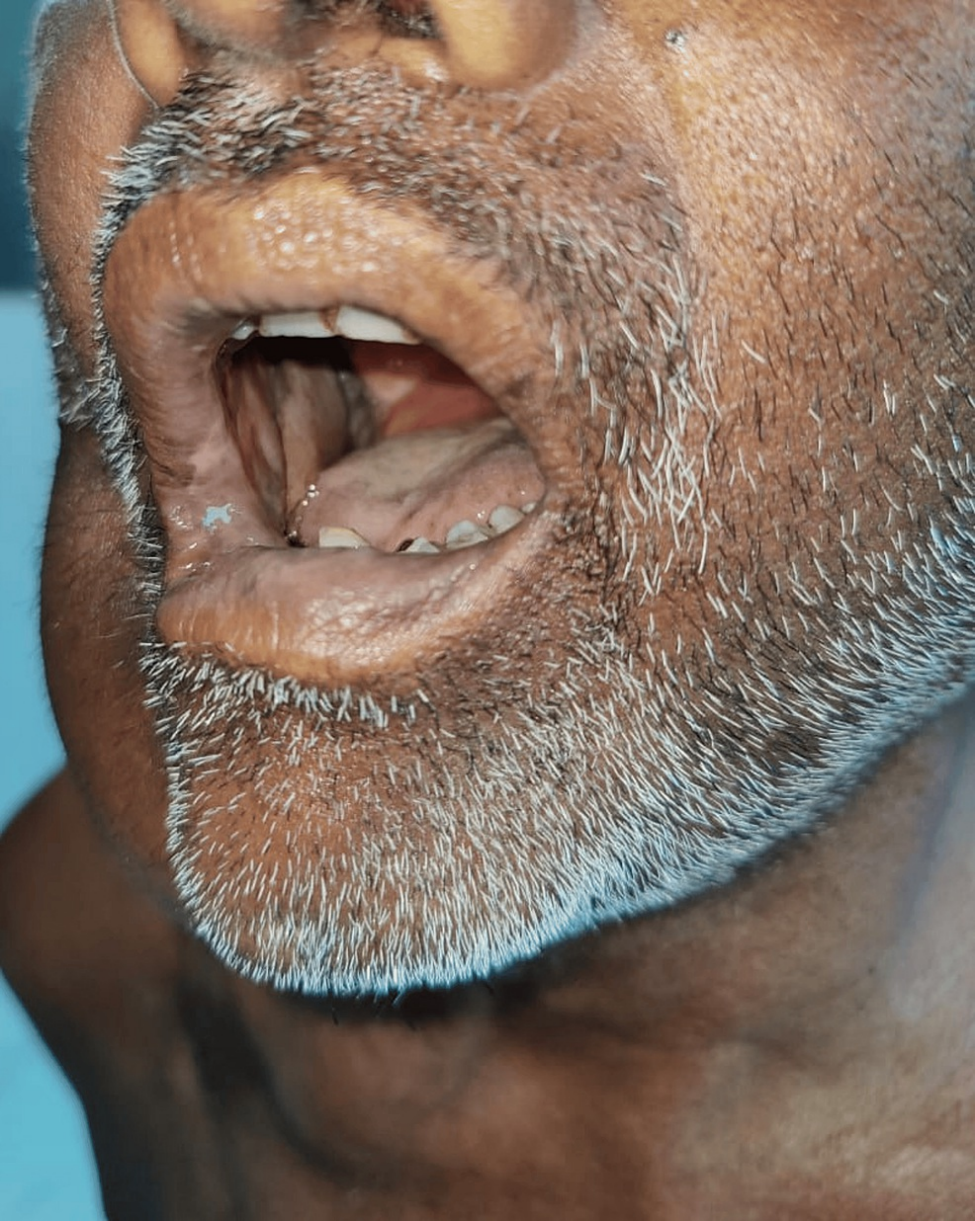
Mucosal lining by Bipaddle PMMC flap. A follow-up image of a patient who underwent a bipaddle PMMC flap procedure shows a healthy flap forming the mucosal lining.

In cases where both outer and inner lining defect coverage were required, the flap marking was further extended horizontally beyond the underlying PM muscle until the anterior axillary line to make a raised bi-paddle flap. In such cases, the horizontal area lateral to the nipple was also included in the flap to provide oral and skin lining (Figures 5-7). The nipple, along with its complex, was excised before in-setting the flap. The PMMC flap that was harvested was subsequently tunneled and retrieved in the neck region before being directed to address the oral defect. Post-operatively, flap monitoring was done by measuring flap glucose levels [6,7].

## Results

**Figure 8:**
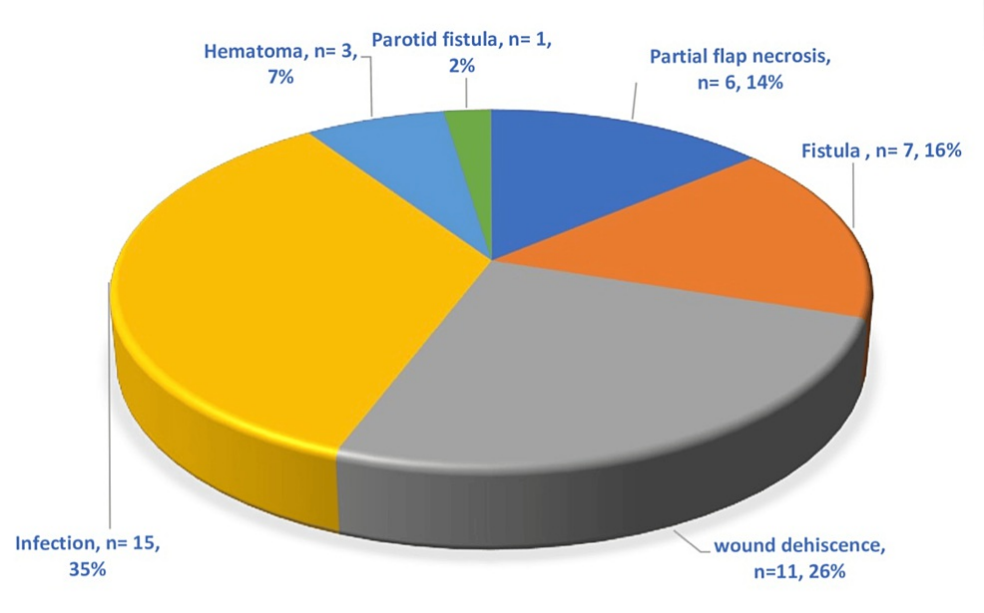
Flap related complications. The total number of patients with complications was 14.

All 48 patients in this study were male and mostly from rural areas. All of them but one had the habit of chewing tobacco and beetles; one patient gave a history of repeated trauma by a sharp tooth without having the habit of tobacco consumption. The mean age of the included patients was 59.07 years. All the tumors were squamous cell carcinoma, and most of them were T4 (TNM classification). The main complaint of the patient at presentation was an ulcer (55%), followed by trismus (32%), and mass in the neck region (26%). Out of 48 PMMC flaps, 12 were designed bi-paddle to cover full-thickness defects involving the skin and mucosa as well. There were no major complications noted in our study. Out of 48 flaps in the study, there was no total flap necrosis and no major flap necrosis as well (Figure 8). The largest PMMC flap harvested for a mucosa was 9 x 6cm, and in the bi-paddle design, it measured 14 x 7cm. There was no wound dehiscence at the donor site in any case. The shift of the Nipple areolar complex (NAC) varied from 1 to 2.5cm in the vertical direction and from 1 to 2 cm in the horizontal direction (Table 1). 

**Table 1:** Demographic profile of patients NA: Not applicable as the nipple was excised while raising bi-paddle flap; RMT: Retromolar trigone

S.No.	Age in years	Site	TNM	Flap size (in cm)	Involvement of skin	Involvement of Mandible	Nipple shifting Vertical (cm)	Nipple shifting Horizontally(cm)
1	45	Buccal mucosa	T4N1	12 x 6	+	-	NA	NA
2	56	Lower alveolus	T4N2a	7 x 4	-	+	2	1
3	70	Lower alveolus	T4N2b	11 x 6	+	+	NA	NA
4	68	Buccal mucosa	T2N0	8 x 5	-	-	2.5	1
5	62	RMT	T4N2b	7 x 5	-	+	2	1
6	52	Buccal mucosa	T4N1	8 x 4	-	+	2	1
7	66	RMT	T4N2a	14 x 7	+	+	NA	NA
8	32	Buccal mucosa	T3N2a	6 x 4	-	-	1.5	1.5
9	58	Buccal mucosa	T2N0	6 x 5	-	-	1.5	1
10	64	Lower alveolus	T4N2a	8 x 5	-	+	2	1.5
11	42	Lower alveolus	T4N2b	8 x 6	-	+	2.5	1.5
12	55	Buccal mucosa	T4N2b	7 x 5	-	+	2.5	1
13	46	RMT	T4N1	8 x 5	-	-	2	1
14	57	Buccal mucosa	T2N2a	6 x 5	-	-	2	1.5
15	69	Buccal mucosa	T2N2a	6 x 4	-	-	1.5	1
16	67	Buccal mucosa	T3N1	9 x 6	-	-	2.5	2
17	62	Lower alveolus	T4N2b	7 x 4	-	+	2	1
18	53	Buccal mucosa	T2N1	7 x 5	-	-	2.5	1.5
19	65	Oral commissure	T4N2a	12 x 6	+	-	NA	NA
20	71	Buccal mucosa	T3N2b	9 x 6	-	-	2	2
21	59	Buccal mucosa	T3N1	8 x 5	-	-	2	1.5
22	64	Lower alveolus	T4N2a	11 x 5	+	+	NA	NA
23	41	Buccal mucosa	T3N2a	8 x 6	-	-	2.5	1
24	56	Buccal mucosa	T4N2a	7 x 5	-	+	2	2
25	43	Buccal mucosa	T4N1	13 x 6	+	-	NA	NA
26	58	Buccal mucosa	T4N2b	9 x 5	-	+	2.5	2
27	72	Buccal mucosa	T4N2b	8 x 6	-	+	2.5	1.5
28	66	Buccal mucosa	T2N0	6 x 4	-	-	1	1
29	60	Buccal mucosa	T4N2b	6 x 5	-	+	1	1.5
30	54	Buccal mucosa	T4N1	8 x 5	-	+	2.5	1
31	64	Lower alveolus	T4N2a	12 x 6	+	+	NA	NA
32	70	Buccal mucosa	T2N2a	7 x 5	-	-	2	2
33	56	Oral commissure	T4N0	11 x 5	+	-	NA	NA
34	66	Buccal mucosa	T4N2a	7 x 6	-	+	1.5	1
35	40	Buccal mucosa	T4N2b	12 x 5	+	-	NA	NA
36	57	Lower alveolus	T4N2b	6 x 4	-	+	1	1.5
37	47	Buccal mucosa	T4N1	13 x 6	+	-	NA	NA
38	58	Buccal mucosa	T2N2a	9 x 6	-	-	2.5	2
39	72	Buccal mucosa	T2N2b	7 x 4	-	-	2	1.5
40	66	Buccal mucosa	T3N0	7 x 5	-	-	2	2
41	64	Buccal mucosa	T4N2b	8 x 5	-	+	1	1.5
42	50	Buccal mucosa	T3N1	8 x 6	-	-	2.5	2
43	64	Oral commissure	T4N2a	11 x 5	+	-	NA	NA
44	70	Buccal mucosa	T2N2a	7 x 5	-	-	2	1.5
45	56	Buccal mucosa	T2N0	7 x 5	-	-	2	2
46	66	Lower alveolus	T4N2a	12 x 6	+	+	NA	NA
47	40	Buccal mucosa	T3N2b	8 x 5	-	-	2	2
48	57	Buccal mucosa	T2N2b	8 x 6	-	-	2.5	1.5

## Discussion

In today's context, microvascular-free flaps are commonly recognized as the top choice for head and neck reconstruction [8,9]. Nonetheless, utilizing these free flaps requires advanced and expensive microvascular equipment, specialized surgical expertise, and rigorous postoperative surveillance.

Since its introduction in 1979 [10], the PMMC technique has stood out as a significant advancement in the history of head and neck reconstruction. This method boasts several advantages: proximity to the head and neck area, provision of well-vascularized tissue, reach extending up to the mid-face, and minimal donor site morbidity.

Additionally, the feasibility of performing PMMC as a single-stage procedure [11] has solidified its position as a widely favored option for head and neck reconstruction. Furthermore, the PMMC flap serves as a valuable salvage option in scenarios where microvascular flaps fail or experience necrosis. It is particularly useful when dealing with patients who have compromised recipient microvasculature due to prior radiotherapy or those who cannot endure extended surgical procedures due to underlying health conditions. The PMMC flap can also be combined with free flaps and presents a more manageable learning curve for novice surgeons adopting the technique.

**Figure 9:**
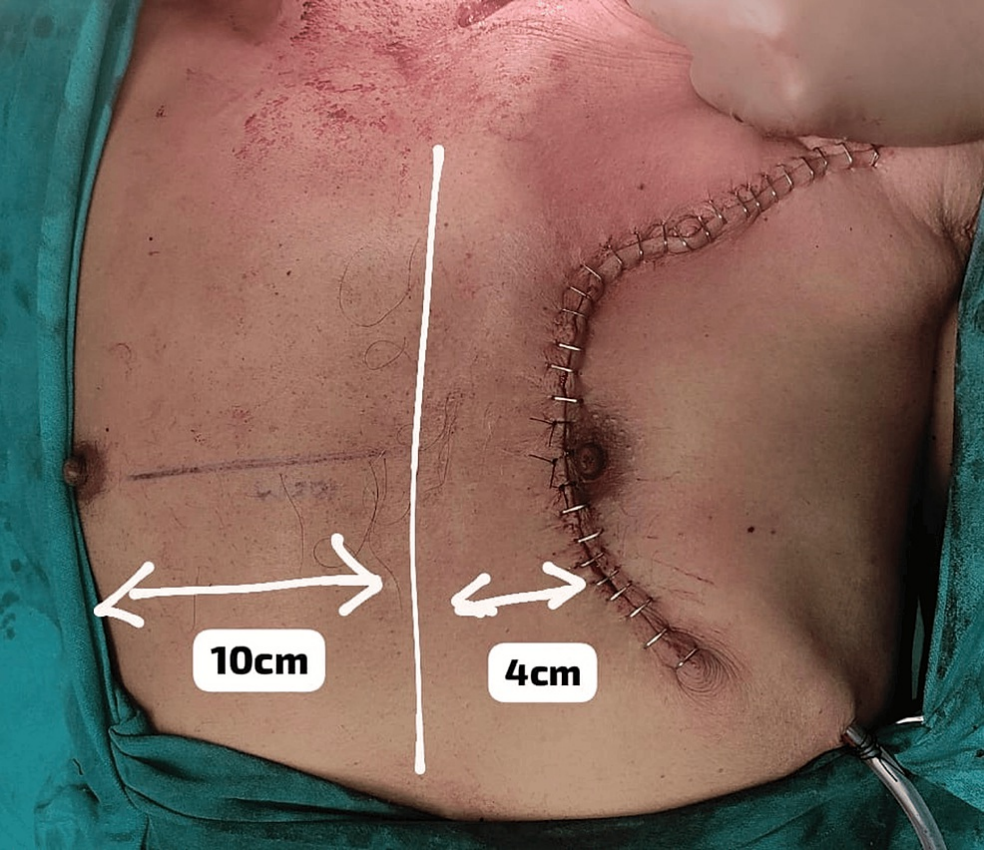
Shift of nipple areolar complex A significant shift was apparent on the left side when PMMC was harvested using conventional techniques.

Based on our prior experiences with conventional skin flap pedicle design, we have observed a significant movement of the NAC towards the medial side (Figure 9). However, in the current study using a rotational advancement flap technique with the lax skin from the chest's lateral side, the maximum observed medial shift of the nipple was only 2 cm, with a vertical shift reaching up to 2.5 cm. Furthermore, we noticed that the extent of this shift increased when the flap's lower edge was further from the NAC in a downward direction and/or with an increase in the width of the flap. Therefore, by tailoring the flap design appropriately, one can regulate the NAC's shifting. This minimal movement is likely due to the strategic use of the relaxed skin from the chest's lateral side, which provides additional skin for a seamless closure of the donor site. Such a tension-free closure significantly reduces complications at the donor site. The observed issue was the development of a standing cone deformity at the base of the rotational flap. This dog ear can be excised later as a minor procedure under local anesthesia. A similar concept of using the lateral part of breast skin was designed by Sandeep M. et al. [12] for female patients to avoid distortion in female patients. 

The existing literature on PMMC flap reconstruction presents varying definitions of complications and their occurrence rates. Complications have been reported to range between 17% and 63% [9,13,14]. In our study, we noted a 30% complication rate. The complications were managed by debridement and secondary suturing in cases of partial necrosis of the flap, evacuation of the hematoma, and dressing and/or antibiotics in cases of infection. Flap necrosis appeared in 6% of cases that were also partial. There was no instance of either total flap loss or major flap necrosis. Our findings do not align with those reported in other studies in terms of total flap failure or major flap necrosis [15-17].

A significant advantage of PMMC flaps lies in their survival rate. Even when performed by a skilled microsurgeon, free flap reconstructions may result in total flap necrosis. However, complete loss of PMMC flaps is rare.

Tonsbeek et al. recently conducted a comparison between pectoralis major myocutaneous and myofascial flap outcomes. They observed a significant difference in suture line leakage but did not find variations in other complications, including donor site complications [18].

Rikimaru et al. emphasized the importance of positioning the skin island medially to the nipple, covering the fourth, fifth, and sixth intercostal spaces. This placement ensures the inclusion of the skin perforator vessels originating from the intercostal branches of the internal thoracic artery, which are supplied by the pectoralis branch of the thoracoacromial artery through open choke vessels during PMMC flap elevation [5].

Consequently, an entirely axial myocutaneous flap can be created by adhering to this anatomical consideration. However, when the flap extends below the seventh rib, the vascular supply shifts to the cutaneous branches of the superior epigastric artery. Including skin portions beyond this boundary in the flap design results in an axial flap with a distal random component, thereby elevating the risk of partial flap loss. While designing the flap, we took into consideration all these aspects. In our study, it's worth noting that the inferior margin of our flap did not extend below the sixth intercostal space in any case, which could potentially explain the absence of major flap loss.

According to our study, the PMMC flap continues to be a preferred method for head and neck reconstruction due to its satisfactory cosmetic and functional results.

Limitations of this study

This retrospective study was conducted at a single center and suffered from a limited sample size with a short follow-up period. The reduced sample size and abbreviated follow-up duration were attributed to the onset of the COVID-19 pandemic, during which hospital visits were significantly curtailed due to lockdown measures.

## Conclusions

Owing to its adaptability, reliable vascular supply, and straightforward learning process, the PMMC flap remains a preferred choice for head and neck reconstruction. This is especially true in areas with constrained resources and high patient demand.

Additionally, by orienting the flap axis horizontally and utilizing the skin from the lateral chest through a rotational advancement flap technique, the extent of nipple-areola complex (NAC) displacement towards the medial side can be minimized.
